# Supplementary material for: The interactome of intact mitochondria by cross-linking mass spectrometry provides evidence for coexisting respiratory supercomplexes
Source: Mol Cell Proteomics. 2017 Dec 8;17(2):216–32. doi: 10.1074/mcp.RA117.000470 (PMC5795388; doi:10.1074/mcp.RA117.000470)
Supplement: Supplemental Data [file supp_RA117.000470_133922_0_supp_23978_fzffwf.zip › spectra_annotation/mito_DR_spectra_annotation/134-1-3-1-6-1.pdf]

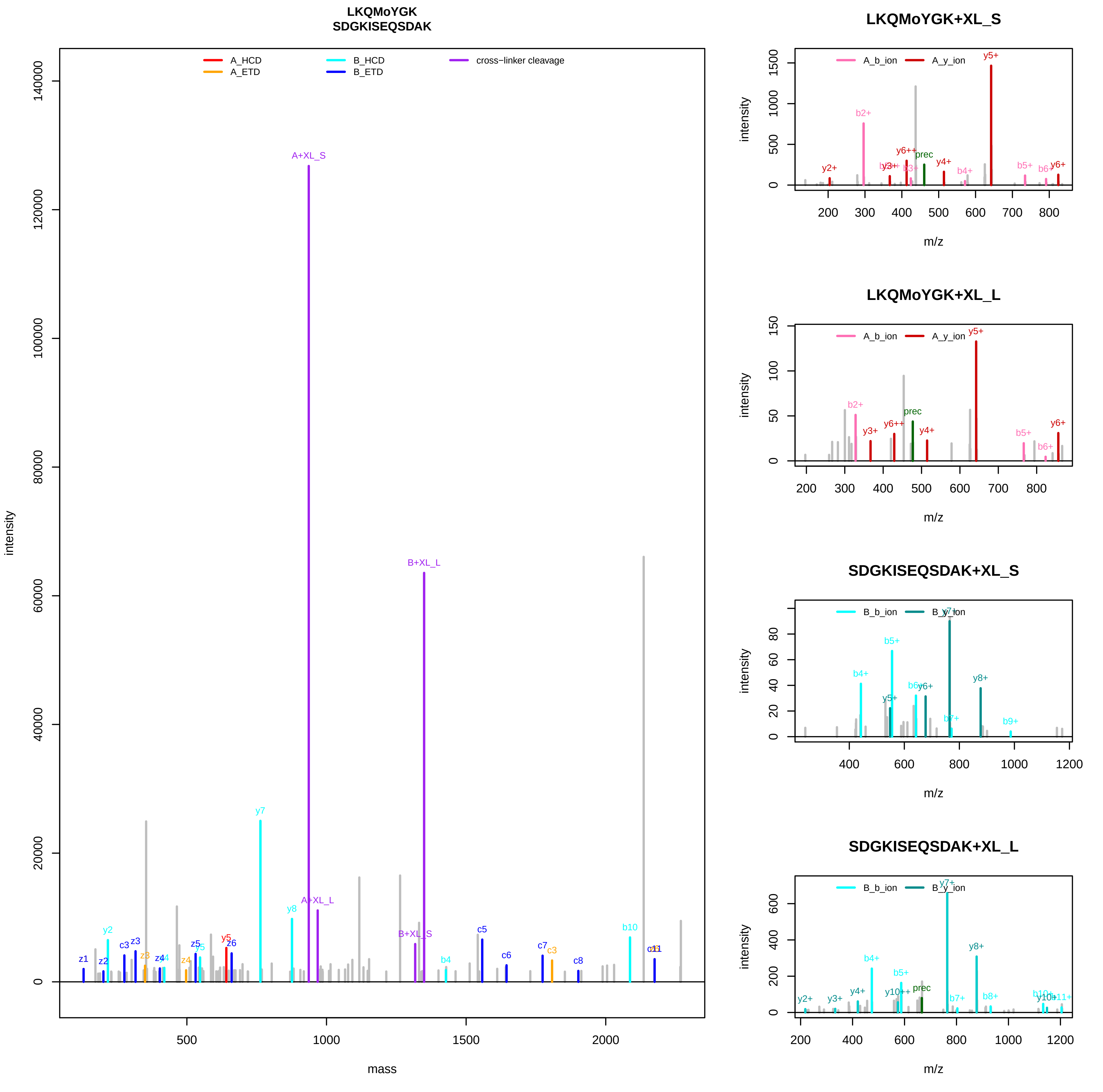
LKQMoYGK
SDGKISEQSDAK
A_HCD
A_ETD
B_HCD
B_ETD
cross−linker cleavage
A+XL_S
B+XL_L
A+XL_L
B+XL_S
intensity
mass
0
20000
40000
60000
80000
100000
120000
140000
500
1000
1500
2000
z1
z2
y2
c3
z3
z3
z4
y4
z4
z5
y5
y5
z6
y7
y8
b4
c5
c6
c7
c3
c8
b10
c11
LKQMoYGK+XL_S
A_b_ion
A_y_ion
y5+
b2+
y6++
prec
y2+
y3+
b3+
y4+
b4+
b5+
b6+
y6+
intensity
m/z
0
500
1000
1500
200
300
400
500
600
700
800
LKQMoYGK+XL_L
A_b_ion
A_y_ion
y5+
b2+
prec
y3+
y6++
y4+
b5+
b6+
y6+
intensity
m/z
0
50
100
150
200
300
400
500
600
700
800
SDGKISEQSDAK+XL_S
B_b_ion
B_y_ion
y7+
b5+
b4+
b6+
y6+
y5+
y8+
b7+
b9+
intensity
m/z
0
20
40
60
80
400
600
800
1000
1200
SDGKISEQSDAK+XL_L
B_b_ion
B_y_ion
y7+
y8+
b4+
b5+
prec
y2+
y3+
y4+
y10++
b7+
b8+
b10+
y10+
b11+
intensity
m/z
0
200
400
600
200
400
600
800
1000
1200
